# Development of Anti-Idiotypic Nanobody-Phage Based Immuno-Loop-Mediated Isothermal Amplification Assay for Aflatoxins in Peanuts

**DOI:** 10.3390/toxins12090565

**Published:** 2020-09-02

**Authors:** Jiawen Lei, Xiaole Han, Xiaoqian Tang, Haiying Wang, Qi Zhang

**Affiliations:** 1 College of Life Sciences, South-Central University for Nationalities, Wuhan 430074, China; jiawenlei@scuec.edu.cn (J.L.); wanghaiying@mail.scuec.edu.cn (H.W.); 2 College of Chemistry and Material Sciences, South-Central University for Nationalities, Wuhan 430074, China; han-xiaole@mail.scuec.edu.cn; 3 Oil Crops Research Institute of the Chinese Academy of Agricultural Sciences, Wuhan 430062, China; tangxiaoqian@caas.cn

**Keywords:** aflatoxin, LAMP, nanobody, immunoassay

## Abstract

Aflatoxin contamination in agricultural products has posed serious health hazards and brought huge economic loss in the food and feed industries. Monitoring aflatoxins in various foods and feeds has become a crucial means to protect public health. This study aimed to report an immuno-loop-mediated isothermal amplification (iLAMP) assay by using an anti-idiotypic nanobody-phage for on-site and rapid detection of aflatoxin in real samples. The iLAMP method was developed on the basis of a competitive immunoassay and LAMP reaction performed in a simple water bath. This method can provide visualized test results: violet color represents positive samples while sky blue represents negative. The visual detection limits of iLAMP for aflatoxin B_1_, B_2_, G_1_, and G_2_ in peanut samples were 1.6, 1.6, 3.2, and 16 μg/kg, respectively. The developed assay was verified with high performance liquid chromatography (HPLC) for the analysis of aflatoxins in peanuts, which demonstrated that the iLAMP method can be applied to the detection of aflatoxin in real samples. The novel iLAMP assay eliminates the need for aflatoxin conjugates, the antibody labeling process, and special equipment, and offers an alternative to existing methods with advantages of time-saving, cost-effectiveness, and ease-of-use.

## 1. Introduction

Aflatoxins are highly toxic secondary metabolites produced by fungal species of *Aspergilli*, especially *Aspergillus flavus* and *Aspergillus parasiticus* [1]. They are listed as group I liver carcinogens by the International Agency for Research on Cancer (IARC) [2]. Till now, more than 20 aflatoxins have been identified, but only Aflatoxins B_1_, B_2_, G_1_, and G_2_ occur naturally in a variety of agricultural foodstuffs, especially peanuts and cereals [3,4]. Aflatoxin contamination poses a serious threat to human health as well as to the food industry, and has received considerable public attention over the past decades [5]. Monitoring aflatoxins in various food and feeds has become a crucial means to protect public health. Currently, many methods have been developed for aflatoxin detection to meet public concerns about food safety and increasingly stringent analytical requirements, ranging from confirmatory tests in official laboratories to rapid on-site tests in the field [6].

As a sensitive, specific and efficient detection method, immunoassay has been widely used in aflatoxin detection, such as enzyme-linked immunosorbent assay (ELISA) [7], immunochromatographic assay (ICA) [8], and immunosensor assay [9]. Nevertheless, all these methods require labelling of antibodies with signal substances (e.g., horseradish peroxidase (HRP), gold nanoparticles (GNPs), and quantum dots (QDs)) to obtain detection signals; the labeling process is often time-consuming and involves tedious covalent coupling chemistry which may result in the loss of antibody activity [10]. Moreover, since aflatoxins are small molecules, they must be coupled to carrier proteins to form aflatoxin conjugates (e.g., AFB_1_-BSA and AFB_1_-OVA) before use in a competitive immunoassay [11]. Researchers either buy the commercial aflatoxin conjugates or synthesize themselves. Both methods are costly, especially the latter, where the synthesis process is complex and unfriendly to operators and the environment [12]. Previously, we have described an immuno-polymerase chain reaction (iPCR) method for aflatoxin detection in grains and feedstuffs [13]. The iPCR assay was based on an anti-idiotypic nanobody-phage which can mimic the antigen instead of aflatoxin conjugates and contains specific DNA sequence that can be easily amplified. As a consequence, there is no need for the aflatoxin conjugates and antibody labeling process in iPCR assay. Although we have applied the iPCR method to the determination of aflatoxins in real samples, the method has some limitations. First, a complete PCR procedure usually lasts 2–3 h, which is time-consuming and not suitable for rapid testing. Second, PCR requires expensive equipment which may not be available in low-tech environments or in the field. Third, the detection results of iPCR cannot be attained directly and must be calculated by a standard curve, which is not applicable for on-site screening. Therefore, a simple, fast, and cost-effective detection method is still needed to compensate for the limitations of the iPCR assay.

Loop-mediated isothermal amplification (LAMP) is an innovative technique for rapid and easy detection of target nucleic acid [14]. Since first reported in 2000, LAMP has been applied in various fields, such as pathogen detection [15], disease diagnosis [16] and genetically modified (GMO) food identification [17]. As an alternative to PCR-based analysis, LAMP has many significant advantages. Firstly, LAMP has higher specificity and efficiency than PCR by using four specially designed primers for six different regions of the target gene, which enables the amplification to be completed within 1 h [18,19,20]. Secondly, LAMP can amplify DNA in isothermal conditions without any thermal cycler by using the *Bst* polymerase enzyme. Thus, the amplification can be performed in a simple heater, for example, a water bath, which makes on-site testing feasible [21,22]. Finally, the results of LAMP can be easily identified by the naked eye without any special instruments by adding the indicator (e.g., SYBR (Synergy Brands, Inc.) Green I) [23,24]. These unique characteristics of LAMP make it an excellent tool to develop rapid, on-site and point-of-care detection methods for aflatoxins. However, there are no reports on the detection of aflatoxins using LAMP.

In this study, we developed an immuno-LAMP (iLAMP) assay for aflatoxin detection based on anti-aflatoxin monoclonal antibody 1C11 (mAb 1C11) and anti-idiotypic nanobody-phage V2–5 specific for mAb 1C11. The novel iLAMP assay eliminates the need for aflatoxin conjugates, antibody labeling process, and special equipment, which offers an alternative to existing methods with advantages of time-saving, cost-effectiveness, and ease-of-use.

## 2. Results

### 2.1. Principle of iLAMP

**Figure 1:**
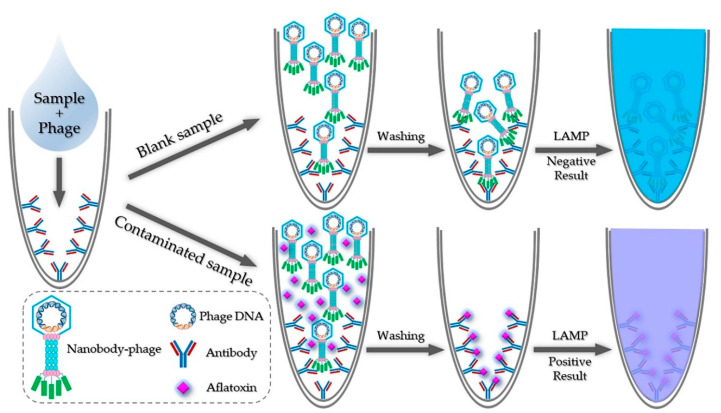
Schematic diagram of the immuno-loop-mediated isothermal amplification (iLAMP). Anti-aflatoxin mAb 1C11 was first coated on the bottom of a polymerase chain reaction (PCR) tube which had been treated with glutaraldehyde. Then, the sample extract and anti-idiotypic nanobody-phage V2–5 were added to the tube simultaneously. Since the nanobody-phage could mimic the antigen (aflatoxin) in the immunoreaction, there was a competition between the phage and aflatoxin in binding with mAb 1C11. After the reaction, the unbound phages were moved out of the tube via the washing step. Subsequently, the LAMP solutions were added to the tube for amplification. The color of the mixture in the tube remained violet (positive result) if the sample contained aflatoxin, otherwise it turned sky blue (negative result).

The principle of iLAMP is shown in Figure 1. The iLAMP method was developed on the basis of a competitive immunoassay and LAMP reaction. Anti-aflatoxin mAb 1C11 [25] was first coated on the bottom of a PCR tube which had been treated with glutaraldehyde. Then, the sample extract and anti-idiotypic nanobody-phage V2–5 were added to the tube simultaneously. Since the nanobody-phage could mimic the antigen (aflatoxin) in the immunoreaction, there was a competition between the phage and aflatoxin in binding with mAb 1C11. After the reaction, the unbound phages were moved out of the tube via the washing step. Subsequently, the LAMP solutions were added to the tube for amplification. The color of the mixture in the tube remained violet (positive result) if the sample contained aflatoxin, otherwise, it turned sky blue (negative result).

### 2.2. LAMP Primers

**Figure 2:**
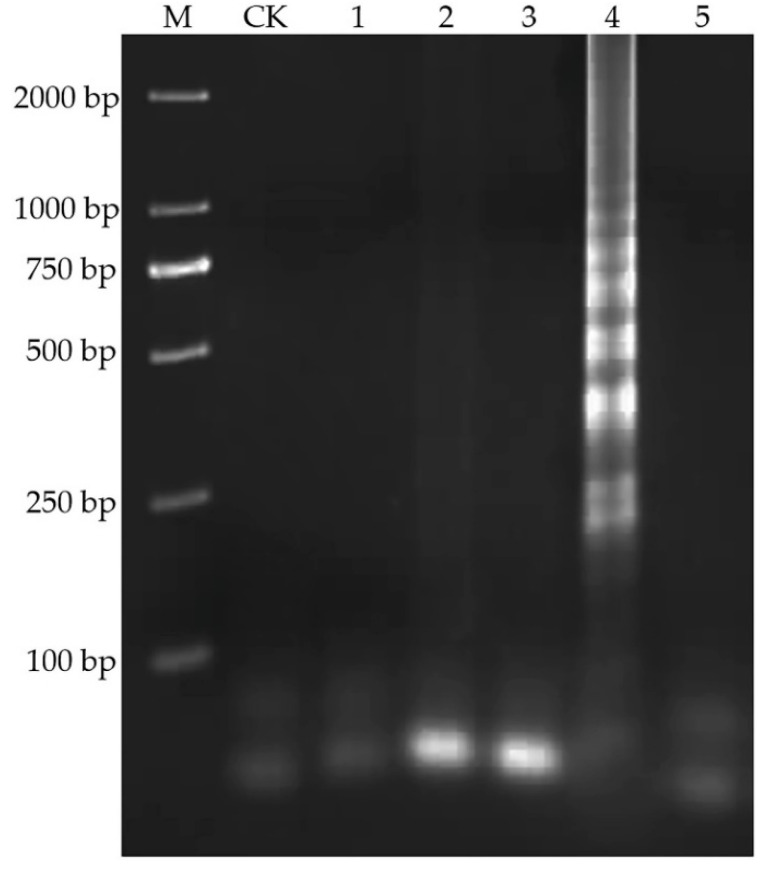
Analysis of LAMP products with five sets of designed primers by agarose gel electrophoresis. Lane M: DNA Marker; Lane CK: ddH2O; Line1–5: 1–5 sets of designed primers.

**Figure 3:**
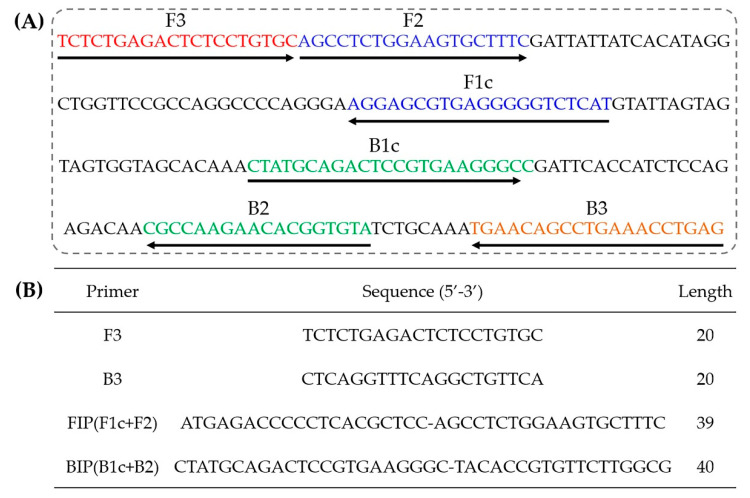
(**A**) Nucleotide sequence of phage V2–5 used to design LAMP primers. The locations and binding sequences of the primers are indicated by arrows and different colors. (**B**) Information of the fourth set of LAMP primers, a forward inner primer (FIP) consisting of F1c and F2, a backward inner primer (BIP) consisting of B1c and B2, a forward outer primer (F3) and a backward outer primer (B3).

Four primers should be designed for six different regions of the target gene: a forward inner primer (FIP) consisting of F1c and F2, a backward inner primer (BIP) consisting of B1c and B2, a forward outer primer (F3) and a backward outer primer (B3). In this research, five sets of LAMP primers based on the DNA sequence of nanobody phage V2–5 were designed by using Primer Explorer V4 online. The five sets of primers were amplified by LAMP with phage V2–5 as template, and ddH_2_O instead of template was added to the reaction system as negative control. The amplification products were analyzed by 2% agarose gel electrophoresis (Figure 2). The results showed that the fourth set of primers had the typical trapezoidal bands amplified by LAMP, while other sets of primers did not, indicating that the fourth set of primers had the specific amplification with the DNA of phage V2–5. Therefore, the fourth set of primers was selected as the specific primers in the follow-up experiment. The priming sites and sequence of LAMP targets are shown in Figure 3A, and information on the primers is shown in Figure 3B.

### 2.3. Specificity of LAMP

**Figure 4:**
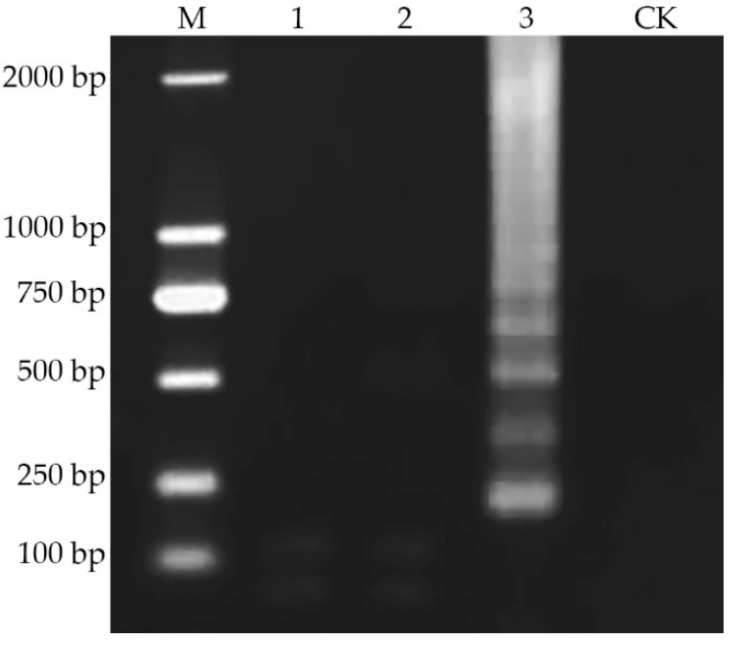
Specificity test of the LAMP Reaction. Specificity was tested by detecting helper phage M13KO7, anti-aflatoxin scFv phage 1A7 and phage V2–5; the results were observed by 2% agarose gel electrophoresis under imaging system. Line M: DNA Marker; Lane CK: ddH2O; Line 1: helper phage M13KO7; Line 2: scFv phage 1A7; Line 3: nanobody phage V2–5.

In order to evaluate the specificity of the LAMP reaction, the helper phage M13KO7, the anti-aflatoxin scFv phage 1A7 [26] and the phage V2–5 were selected as templates for LAMP reaction, and ddH_2_O instead of the template was used as the negative control. Each amplification product was subjected to 2% agarose gel electrophoresis (Figure 4). The results showed that only the nanobody-phage V2–5 produced trapezoid bands, while the other three phages did not, indicating that the primers have good specificity and can be used for the specific LAMP reaction of nanobody phage V2–5.

### 2.4. Visual Inspection of LAMP

**Figure 5:**
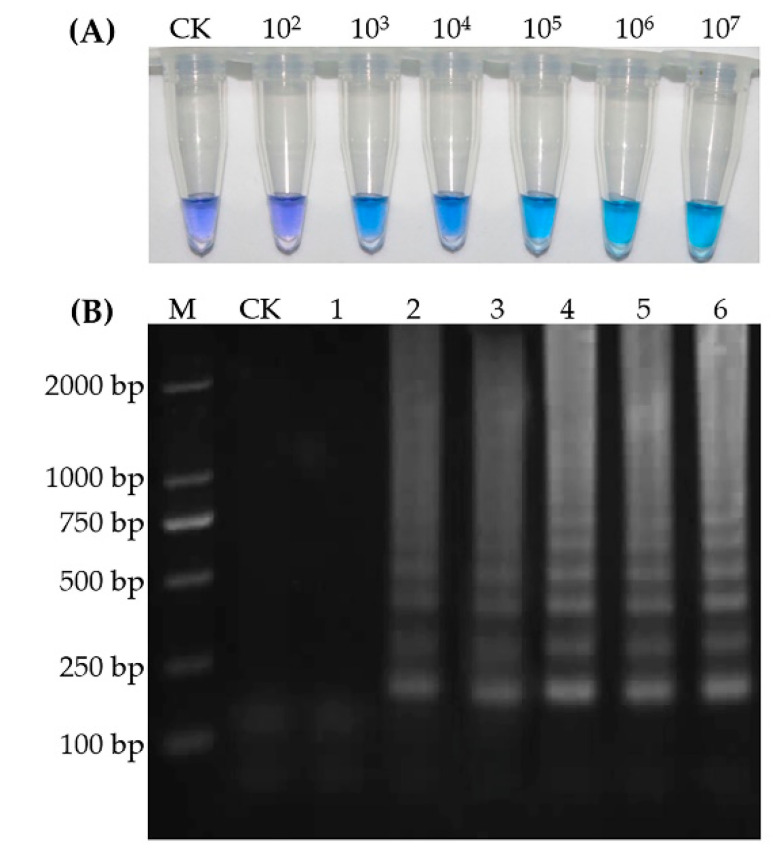
(**A**) Visual Inspection result of LAMP. The color change of the mixture in the tubes was observed by the naked eye. (**B**) Agarose gel electrophoresis result of the LAMP products. M represents DNA Marker; CK represents negative control; 1–6 represents 10^2^, 10^3^, 10^4^, 10^5^, 10^6^ and 10^7^ pfu/mL phage V2–5, respectively.

By adding an indicator to the LAMP system, the detection results can be identified by the naked eye easily. The indicator used in this study was hydroxy-naphthol blue (HNB), a metal ion indicator. When HNB was first added to the reaction system, the color of the mixture was violet; during the reaction, Mg^2+^ combined with the pyrophosphate ion, a by-product of LAMP, to form magnesium pyrophosphate precipitation; meanwhile, the color of the mixture changed from violet to sky blue with the decrease of Mg^2+^ concentration. In order to investigate whether the visual results were consistent with those of DNA gel electrophoresis, phage V2–5 was diluted into concentration gradients of 10^2^, 10^3^, 10^4^, 10^5^, 10^6^ and 10^7^ pfu/mL, and detected by LAMP, respectively. The visual results are shown in Figure 5A, and the DNA gel electrophoresis results are shown in Figure 5B. The detection limits of phage V2–5 obtained by the two methods were both 10^3^ pfu/mL. Therefore, the visual detection method can be used instead of DNA gel electrophoresis to simplify the detection process.

### 2.5. Visual Detection Limit of iLAMP

**Figure 6:**
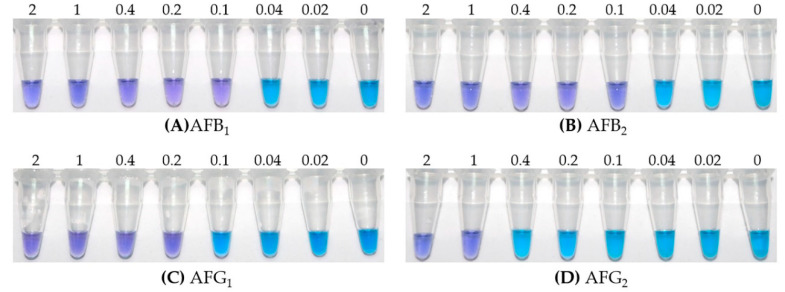
Visual detection limits of iLAMP for four aflatoxins. A series of concentrations (2, 1, 0.4, 0.2, 0.1, 0.04 and 0.02 µg/kg) of aflatoxin standards were prepared in 10% methanol/PBS (v/v) and detected by iLAMP method; a sample without aflatoxin was used as blank control (0 µg/kg). (**A**) Visual detection limit of AFB_1_, (**B**) Visual detection limit of AFB_2_, (**C**) Visual detection limit of AFG_1_, (**D**) Visual detection limit of AFG_2_.

A series of concentrations (2, 1, 0.4, 0.2, 0.1, 0.04 and 0.02 µg/kg) of aflatoxin standards were prepared in 10% methanol/PBS (v/v) and detected by the iLAMP method. The color change of the tubes was observed with the naked eye. As shown in Figure 6, with the decrease of aflatoxin concentration, the color of the tubes gradually changed from violet to sky blue. The lowest concentration of aflatoxin under violet color was taken as the visual detection limit for iLAMP. Therefore, the visual detection limits of iLAMP for AFB_1_, AFB_2_, AFG_1_ and AFG_2_ were 0.1, 0.1, 0.2 and 1 µg/kg, respectively.

### 2.6. Solvent and Matrix Effects

Due to their low solubility in water, aflatoxins are usually extracted from samples by high concentrations of methanol. In real sample detection, the binding of antibody and antigen is often interfered with by either methanol or sample matrix in the sample extract. Dilution is a common and simple way to eliminate these effects [12].

**Figure 7:**
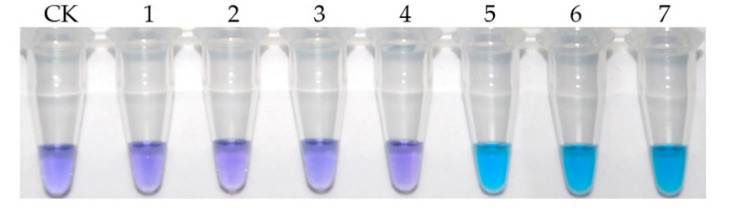
Effects of sample extract dilution ratio to the reaction between mAb 1C11 and phage V2–5. CK represented negative control without sample extract and phage V2–5; 1 represented undiluted sample extract; 2–6 respectively represented 2, 4, 8, 16, and 20 times dilution of the sample extract; 7 represented positive control with PBS and phage V2–5.

The extract of blank peanut sample was diluted with PBS by 2, 4, 8, 16, and 20 times. The phage V2–5 was mixed with an equal volume of each sample dilution and detected by the iLAMP method. The color of the tube under each dilution ratio was observed with the naked eye, and the results are shown in Figure 7. When the sample extract was diluted 2, 4 and 8 times, the color of the tubes were the same as the blank control (violet), indicating that the sample extract affected the binding of mAb 1C11 and phage V2–5. When the extract was diluted more than 16 times, the amplification began, indicating that the effect of the sample extract can be ignored. Since a higher dilution ratio would reduce the sensitivity of the method, 16 times was chosen as the best dilution ratio of the sample extract.

### 2.7. Assay Validation with Peanut Samples

To validate the assay, 20 naturally contaminated peanut samples were analyzed by iLAMP and high performance liquid chromatography (HPLC) methods, respectively. The results obtained by the two methods are shown in Table 1. The visual inspection results of iLAMP were consistent with those from HPLC. The five replications within the iLAMP assay also showed good repeatability, demonstrating that the newly developed iLAMP method can be applied to the determination of aflatoxin in real samples.

### 2.8. Comparison of iLAMP with Previous Research

Based on mAb 1C11, we have previously established a variety of immunoassays for aflatoxins, which can be divided into two categories: methods using aflatoxin conjugates (ELISA and ICA), and methods using nanobody instead of conjugates (nanobody-based ELISA and nanobody-phage-based real-time iPCR). The comparison of assay parameters and analytical characteristics between iLMAP and previous research is shown in Table 2. Obviously, the methods with aflatoxin conjugates were more sensitive (in AFB_1_ detection). However, it is more environmentally friendly, safer for operators, and cost effective to use nanobody instead of the conjugates. Compared with the other two methods using nanobodies, the iLAMP established in this work has the highest sensitivity, and also achieves rapid detection in the field.

## 3. Discussion

### 3.1. Primer Design of LAMP

LAMP primers should be designed in accordance with specific requirements in addition to general PCR primer design principles. For example, the target gene sequence for primer design should be less than 22kbp. Furthermore, the annealing temperature of primers should be between 60–65 °C, which was also the optimal temperature for *Bst* DNA polymerase. In this study, five sets of primers were designed by LAMP online primer design software, and a set of effective primers was obtained after screening. In addition, it has been reported that the LAMP reaction efficiency could be improved by adding two Loop Primers (Loop Primer F and Loop Primer B) to the LAMP system, and the reaction time could be shortened to half of the original [28]. Therefore, loop primers can be utilized in our following study to shorten the reaction time and further simplify the detection process.

### 3.2. Visual Inspection Indicator of LAMP

By adding an indicator to LAMP system, the detection results can be determined by the naked eye, which eliminates the tedious procedures of DNA gel electrophoresis. The most widely used indicator in LAMP is SYBR Green I, which is a highly sensitive DNA fluorescent dye. The LAMP reaction mixture will turn green after the addition of SYBR Green I if there is specific amplification; otherwise, the mixture will remain orange. However, the disadvantage of SYBR Green I is that it must be added to the tube after LAMP reaction. By opening the lid and adding the indicator, the probability of contamination will be greatly increased, which may lead to false positive or negative results. The indicator HNB used in this study can be directly added to the LAMP system before the reaction. Thus, there is no need to open the lid after the reaction, which simplified the operation process and kept the whole detection process within a closed environment; more importantly, the probability of contamination was reduced.

### 3.3. Solvent and Matrix Affects

In this study, aflatoxins were extracted by 80% methanol/water (v/v); as a result, the activity of mAb 1C11 was interfered with by methanol and sample matrix in the sample extract. Eventually, the solvent and matrix effects were eliminated by dilution of the sample extract. However, the sensitivity of iLAMP is lower than the immunochromatographic assay previously established in our laboratory [27]. The possible reason is that the dilution ratio of iLAMP (16 times) is higher than that of the immunochromatographic method (7.5 times). This is also consistent with the reports that a high dilution ratio will reduce the sensitivity of the method [29]. After dilution, the detection limits of iLAMP for aflatoxin B_1_, B_2_, G_1_ and G_2_ in real samples were 1.6, 1.6, 3.2 and 16 μg/kg, respectively.

### 3.4. Application Prospect of iLAMP

As an improved nucleic acid amplification method with advantages of high specificity, and fast and simple operation, LAMP is widely used in detection of bacteria [30], viruses [31] and genes [32], while its application in small molecule detection is rarely reported. In this study, LAMP was applied to the detection of aflatoxin for the first time by using anti-idiotypic nanobody phage V2–5. Compared with immunochromatography assay which also adopts the visual detection method, this method does not need complex procedures such as antibody labeling and test strip assembling. In addition, the iLAMP can be applied for AFB_1_ detection according to the maximum residue limits (MRL) for peanuts in most countries (e.g., 20 μg/kg in China and USA, 10 μg/kg in Japan). According to the MRL of total Aflatoxins (15 μg/kg) set for peanut by Codex, the iLAMP is also applicable since the proportion of four aflatoxins (B_1_, B_2_, G_1_ and G_2_) is generally 1:0.1:0.3:0.03 when they occur simultaneously [3]. In conclusion, the iLAMP method established in this study is fast and simple, does not rely on expensive equipment, and has low operational requirements for operators. This study provides a new method for aflatoxin immunoassay, which can be applied to on-site rapid screening of real samples, and is expected to be popularized and applied in situations lacking instruments and equipment.

## 4. Materials and Methods

### 4.1. Materials

Anti-aflatoxin mAb 1C11 and anti-idiotypic nanobody phage V2–5 specific for mAb 1C11 were produced in our laboratory as previously described. Aflatoxin B_1_, B_2_, G_1_, and G_2_ standard solutions, polyethylene glycol 8000 (PEG8000), Tween-20, betaine, bovine serum albumin (BSA), and hydroxy naphthol blue (HNB) were purchased from Sigma-Aldrich (Merck KGaA, Darmstadt, Germany). The helper phage M13KO7, *E. coli* ER2738, and *Bst* DNA polymerase were purchased from New England Biolabs (Ipswich, MA, USA). dNTPs were purchased from Takara (Kyoto, Japan). The water was produced by a Milli-Q purification system (Merck KGaA, Darmstadt, Germany). The iLAMP method was validated with an Agilent 1100 HPLC system (Santa Clara, CA, USA).

### 4.2. Phage Preparation

The recombinant *E. coli* ER2738 containing nanobody V2–5 phagemid were inoculated into 1 mL ampicillin-SB (100 μg/mL) medium, with shaking (225 rpm) at 37 °C overnight. The 1 mL overnight culture of *E. coli* was added to 100 mL ampicillin-SB (100 μg/mL) medium, followed by shaking (250 rpm) at 37 °C until OD_600_ reached 0.5−0.6. After being infected with helper phages M13K07(10^11^–10^12^ pfu/mL) by incubation at 37 °C for 30 min, kanamycin was added into the culture at a 70 μg/mL final concentration, followed by shaking (225 rpm) overnight at 37 °C. The overnight culture was centrifuged (10,000 rpm) for 15 min at 4 °C, and the supernatant was mixed with 20% PEG/NaCl by incubation on ice for 2 h. The mixture was centrifuged (10,000 rpm) for 30 min and the precipitate (phage particles) was resuspended with 2 mL of BSA/PBS (PBS containing 0.5% BSA), followed by centrifuging at 12,000 rpm for 5 min. The supernatant was filtered with a 0.22 μM filter and mixed with an equal volume of sterilized glycerin. The phages were stored at −20 °C after titer measurement.

### 4.3. Primer Design

The specific LAMP primers based on the DNA sequence of the anti-idiotypic nanobody-phage V2–5 were designed by an online primer designing tool named Primer Explorer V4 (http://primerexplorer.jp/elamp4.0.0/index.html) developed by Eiken Chemical Co., Ltd. (Tochigi, Japan).

### 4.4. LAMP Reaction

The LAMP mixture contained 1.6 μM each of FIP and BIP, 0.2 μM each of F3 and B3, 1.4 mM dNTPs, 0.8 M betaine, 8 mM MgSO4, 20 mM Tris-HCl (pH 8.8), 10 mM KCl, 10 mM (NH_4_)_2_SO_4_, 0.1% Tween-20, 8U of *Bst* DNA polymerase, 150 μM HNB, 1 μL of target DNA and ddH_2_O in a final volume of 25 μL. The reaction was performed in 0.2 mL PCR tubes which placed in a water bath at 63 °C for 40 min, and then 95 °C for 2 min to terminate the amplification. The tube contained all the LAMP reagents except the DNA template was set as the negative control. The results of LAMP were identified either by naked eye or DNA gel electrophoresis.

### 4.5. iLAMP Method

The PCR tubes were treated using a reported method [33]. 20 μL of 0.8% glutaraldehyde in Milli-Q water was added to 0.2 mL PCR tubes followed by 5 h incubation at 37 °C. After six times washing with Milli-Q water, the tubes were coated with 20 μL of mAb 1C11(10 µg/mL) in carbonate buffer (0.05 M, pH 9.6) at 4 °C overnight. Then, the tubes were blocked with 3% (w/v) skimmed milk-PBS by incubation for 1.5 h at 37 °C. A series concertation of aflatoxin standards prepared in 10% (v/v) methanol/PBS was mixed with an equal volume of phage V2–5 (10^7^ pfu/mL). 20 μL of each mixture was added into the PCR tube and incubated at 37 °C for 1 h. The tubes were washed six times with PBST (PBS with 0.05% Tween-20) followed by six washings with Milli-Q water in order to remove the unbound phages. The phages captured by mAb 1C11 were used as DNA template and detected by LAMP.

### 4.6. Sample Analysis

The peanut samples obtained from local markets were ground by a grinder and stored at −20 °C before use. 5 g of each sample was extracted by 25 mL of 80% methanol/water (v/v) and incubated at room temperature with shaking (250 rpm) for 15 min. Subsequently, the mixture was centrifuged at 5000 rpm for 5 min at room temperature, and the supernatant was further used for sample analysis. The solvent and matrix effects were tested by blank samples. A comparative study was carried out by both iLAMP and HPLC for the validation of the iLAMP method. The aflatoxin purification procedure of HPLC used an immunoaffinity column (IAC) which was made by our laboratory as previously described [34]. The HPLC system was equipped with a 150 × 4.6 mm C_18_ column and a fluorescence detector. The excitation and emission wavelengths were respectively set as 360 and 440 nm. In the mobile phase, acetonitrile and water performed at a flow rate of 1.0 mL/min. The injection volume was fixed as 20 μL.

## Figures and Tables

**Table 1:** Results of high performance liquid chromatography (HPLC) and iLAMP for aflatoxins in peanut samples.

Sample	HPLC (µg/kg)				iLAMP (*n* = 5)
	AFB_1_	AFB_2_	AFG_1_	AFG_2_	
1	3.39	0.20	ND ^a^	ND	V ^b^, V, V, V, V
2	3.73	0.36	ND	ND	V, V, V, V, V
3	2.27	0.22	ND	ND	V, V, V, V, V
4	23.60	3.60	ND	ND	V, V, V, V, V
5	2.56	0.31	ND	ND	V, V, V, V, V
6	12.41	2.30	ND	ND	V, V, V, V, V
7	0.78	ND	ND	ND	S ^c^, S, S, S, S
8	4.90	0.61	ND	ND	V, V, V, V, V
9	1.97	ND	ND	ND	V, V, V, V, V
10	20.68	1.66	ND	ND	V, V, V, V, V
11	1.02	ND	ND	ND	S, S, S, S, S
12	6.24	1.02	ND	ND	V, V, V, V, V
13	16.43	1.92	2.36	ND	V, V, V, V, V
14	0.94	ND	ND	ND	S, S, S, S, S
15	16.84	3.32	ND	ND	V, V, V, V, V
16	28.86	1.30	ND	ND	V, V, V, V, V
17	2.24	0.44	ND	ND	V, V, V, V, V
18	15.88	2.25	ND	ND	V, V, V, V, V
19	5.18	0.66	ND	ND	V, V, V, V, V
20	0.34	ND	ND	ND	S, S, S, S, S

^a^ ND: not detectable. ^b^ V: violet, positive result. ^c^ S: sky blue, negative result.

**Table 2:** Comparison between iLAMP and previous research.

Method	Immune Reagents	Matrix	Sensitivity (µg/kg)	Reference
ELISA	1C11 + AFB_1_-BSA	Standard solution	0.0012	[25]
ICA	1C11 + AFB_1_-OVA	Peanut	0.03	[27]
ELISA	1C11 + Nanobody	Peanut, corn, rice	13.8	[12]
iPCR	1C11 + Nanobody-phage	Peanut, corn, rice, feedstuff	5.6	[13]
iLAMP	1C11 + Nanobody-phage	Peanut	1.6	_ ^a^

^a^: The data were obtained in this work.
